# Comparative Genomic Characterization of Buffalo Fibronectin Type III Domain Proteins: Exploring the Novel Role of FNDC5/Irisin as a Ligand of Gonadal Receptors

**DOI:** 10.3390/biology10111207

**Published:** 2021-11-19

**Authors:** Siwen Wu, Faiz-ul Hassan, Yuhong Luo, Israr Fatima, Ishtiaq Ahmed, Awais Ihsan, Warda Safdar, Qingyou Liu, Saif ur Rehman

**Affiliations:** 1 State Key Laboratory for Conservation and Utilization of Subtropical Agro-Bioresources, Guangxi University, Nanning 530005, China; siwenwu123@163.com (S.W.); luoyuhong0720@163.com (Y.L.); 2 Institute of Animal and Dairy Sciences, University of Agriculture, Faisalabad 38040, Pakistan; f.hassan@uaf.edu.pk; 3 Department of Bioinformatics and Biotechnology, Govt. College University, Faisalabad 38000, Pakistan; fatimaisrar926@gmail.com; 4 School of Medical Science, Gold Coast Campus, Griffith University, Southport, QLD 4222, Australia; ishtiaq.ahmed@griffithuni.edu.au; 5 Department of Biosciences, COMSATS University Islamabad, Sahiwal Campus, Sahiwal 57000, Pakistan; awais@cuisahiwal.edu.pk; 6 Department of Biochemistry, Bahauddin Zakariya University, Multan 60000, Pakistan; wardakhanw9@gmail.com

**Keywords:** buffalo, FN-III proteins, FNDC5/irisin, receptors, binding affinities

## Abstract

**Simple Summary:**

A total of 29 fibronectin type III domain (FN-III) protein genes from the buffalo genome were detected and characterized. The molecular and evolutionary analysis demonstrated the well-conserved nature of FN-III proteins with a variety of stable to unstable, hydrophobic to hydrophilic, and thermostable to thermo-unstable properties. For the first time, we predicted the binding scores and interface residues of FNDC5/irisin as a ligand for six representative receptors, having a functional role in energy homeostasis, and significant involvement in folliculogenesis, and spermatogenesis in buffalo.

**Abstract:**

FN-III proteins are widely distributed in mammals and are usually involved in cellular growth, differentiation, and adhesion. The FNDC5/irisin regulates energy metabolism and is present in different tissues (liver, brain, etc.). The present study aimed to investigate the physiochemical characteristics and the evolution of FN-III proteins and FNDC5/irisin as a ligand targeting the gonadal receptors including androgen (AR), DDB1 and CUL4 associated factor 6 (DCAF6), estrogen-related receptor β (ERR-β), estrogen-related receptor γ (ERR-γ), Krüppel-like factor 15 (KLF15), and nuclear receptor subfamily 3 group C member 1 (NR3C1). Moreover, the putative role of irisin in folliculogenesis and spermatogenesis was also elucidated. We presented the molecular structure and function of 29 *FN-III* genes widely distributed in the buffalo genome. Phylogenetic analysis, motif, and conserved domain pattern demonstrated the evolutionary well-conserved nature of FN-III proteins with a variety of stable to unstable, hydrophobic to hydrophilic, and thermostable to thermo-unstable properties. The comparative structural configuration of FNDC5 revealed amino acid variations but still the FNDC5 structure of humans, buffalo, and cattle was quite similar to each other. For the first time, we predicted the binding scores and interface residues of FNDC5/irisin as a ligand for six representative receptors having a functional role in energy homeostasis, and a significant involvement in folliculogenesis and spermatogenesis in buffalo.

## 1. Introduction

Buffalo (*Bubalus bubalis*) is a unique livestock species with peculiar productive performance, predominantly found in Asia including China, India, and Pakistan [1,2]. Buffaloes are renowned for their unique ability to consume roughages and convert them into valuable products such as meat and milk. Additionally, buffalo can tolerate harsh weather conditions, perform better under poor feeding resources, and provide draught power [3]. Buffalo milk is relished owing to its peculiar taste with a higher protein, fat, and solid content [4,5,6]. Mainly, buffalo in the Mediterranean and South-Eastern region of Asia serves as an important economic component in the agriculture sector [1,7,8]. Despite having excellent production potential, the productivity of the buffalo is jeopardized due to its poor reproductive efficiency. Buffalo as a dairy animal is known as a poor breeder mainly due to major challenges such as a higher rate of infertility, poor estrus expression [9], poor reproductive efficiency [10], distinct seasonal reproductive pattern [11,12], delayed sexual maturity, prolonged calving intervals [13], and low calf survival rates [14]. It is challenging to improvise the buffalo reproductive and energy metabolism efficiency through finding some biological molecular chaperon that could target reproduction-related signaling receptors to improve the reproductive ability of the buffalo.

In animals, fibronectin proteins are widely dispersed in an extracellular matrix with a variety of functions including cellular growth, migration, differentiation, and adhesion. These proteins are involved in important processes such as healing and the replacement of damaged tissues and embryogenesis [15,16].

In large mammals, the regulation of energy homeostasis under metabolic shifts is the foremost challenge to keep normal physiological and molecular functioning [17]. Fibronectin type III domain containing 5 (FNDC5) was initially designated as a critical factor that causes cellular differentiation of skeletal muscle. Principally, it was detected in peroxisomes [18]. Irisin is a myokine involved in higher energy expenditure through stimulation of white adipose tissues. Firstly, the irisin hormone proteolytically dissociates from its precursor FNDC5, which enhances the circulating irisin levels, subsequently reducing insulin resistance while improving glucose homeostasis [19]. Irisin is mainly secreted from subcutaneous, visceral adipose tissue and skeletal muscles [20,21], but a recent study also reported its presence in other tissues including the spleen, liver, brain, stomach, and testis [22]. The regulatory, molecular, and physiological role of FNDC5/irisin has not yet been fully described and various contradictory findings have been documented in this regard. Thus, there is a dire need to explore the mechanism of FNDC5/irisin functioning in mammals.

There is no information available on the structural and functional role of FN-III proteins in the buffalo. Keeping in view the physiological roles of FN-III proteins (particularly FNDC-5), it is imperative to characterize these proteins at the genomic level to better understand their structure and putative functions in the buffalo. This study aimed to investigate the gene structure, physicochemical characteristics, and the evolution of FN-III proteins in buffalo. Additionally, we also explored the putative affinity of FNDC5/irisin as a ligand with key gonadal receptors including AR, DCAF6, ERR-β, ERR-γ, KLF15, and NR3C1, to investigate its possible role in folliculogenesis and spermatogenesis.

## 2. Materials and Methods

### 2.1. Identification of FN-III Family Genes and Characterization of Their Physiochemical Properties

The human and cattle FN-III protein sequences were obtained from NCBI (https://www.ncbi.nlm.nih.gov/ accessed on 3 March 2021) [23] and were used as queries for buffalo FN-III protein sequences identification (Accession numbers are given in Table S1). The human and cattle sequences were searched in the NCBI database to retrieve non-redundant buffalo protein sequences of the FN-III with an E value ≤ 1.0 × e^−5^. Furthermore, the annotated buffalo genome GFF file was used to check the chromosomal position and corresponding location of the *FN-III* genes in the MCScanX program [24]. The physiochemical properties of buffalo FN-III proteins were determined by using the ProtParam tool including the aliphatic index (AI), molecular weight (MW), isoelectric point (pI), grand average of hydropathicity (GRAVY), instability index (II), and the number of amino acids [25]. Moreover, the Multiple Expectation Maximization for Motif Elicitation (MEME) program was used to find a maximum of 10 conserved protein motifs of buffalo FN-III proteins (http://meme-suite.org/tools/meme accessed on 10 March 2021) [26]. Additionally, the conserved domains of buffalo FN-III proteins were confirmed using the NCBI CDD (conserved domain) database (https://www.ncbi.nlm.nih.gov/Structure/cdd/wrpsb.cgi accessed on 3 March 2021) [23].

### 2.2. Collinearity and Multiple Sequence Alignment Analysis

For collinearity analysis, the genome annotated files of cattle and buffalo were used as input files in TBtool to compare the respective gene positions [5]. Three-dimensional structure of FNDC5/irisin protein of human, buffalo, and cattle breeds were designed with Phyre2 software, and fold recognition end homology modeling was used for the identification of secondary structure features (http://www.sbg.bio.ic.ac.uk/phyre2 accessed on 3 March 2021). The multiple sequence alignment of irisin peptides of humans, buffalo, and cattle breeds conducted to visualize the sequence variation and indels in Multiple Align Show (https://www.bioinformatics.org/sms/multi_align.html accessed on 3 March 2021).

### 2.3. Molecular Docking Analysis

Moreover, in order to study the FNDC5/irisin interaction as a ligand with six selected receptors (AR, DCAF6, ERR-β, ERR-γ, KLF15, and NR3C1), the PDB database was searched to retrieve the PDB structures of corresponding proteins. Their respective proteins PDB IDs are 2am9, 3I8E, 4ZI1, 2E2R, 2EBT, and 4p6x, respectively [27]. The HDOCK docking protocol was used for protein–protein interaction to predict the docking scoring of the ligand–receptor complex [28]. The binding interactions of the protein ligand–receptor complex were determined by using ligplot [29].

## 3. Results

### 3.1. Identification of FN-III Gene Family and Their Physiochemical Properties

In this study, a comprehensive strategy was applied to characterize the *FN-III* gene family in the buffalo genome. A total of 29 *FN-III* genes, widely distributed over different chromosomes of buffalo, harboring variable exons, were detected by using cattle and human as a query sequence and their physiochemical features are presented in Table 1. The FN-III protein isoform’s functional diversity in buffalo was realized from their total number of amino acids ranging from 205 (FNDC5) to 3490 (IGFN1) and MW ranged between 20 kDa and 258 kDa (Table 1). Moreover, according to the instability index, all the members of the FN-III family are unstable except FANK1, LRFN5, IGFN, FLRT1, and FLRT2 which are stable. The isoelectric point indicated that most of the FN-III proteins are acidic (pI < 7), while basic FN-III proteins were also found (pI > 7), as shown in Table 1. Additionally, all of the FN-III proteins have AI values greater than 65 exhibiting thermostable abilities except IGFN1 and FNDC1 having lower AI values (<65), which are seen as thermo-unstable proteins. Furthermore, all of the FN-III proteins behaved as hydrophilic owing to their lower GRAVY values, but FNDC7 and FNDC10 were hydrophobic in nature due to their higher GRAVY values (Table 1).

### 3.2. Gene Structure and Motif Analysis

**Figure 1:**
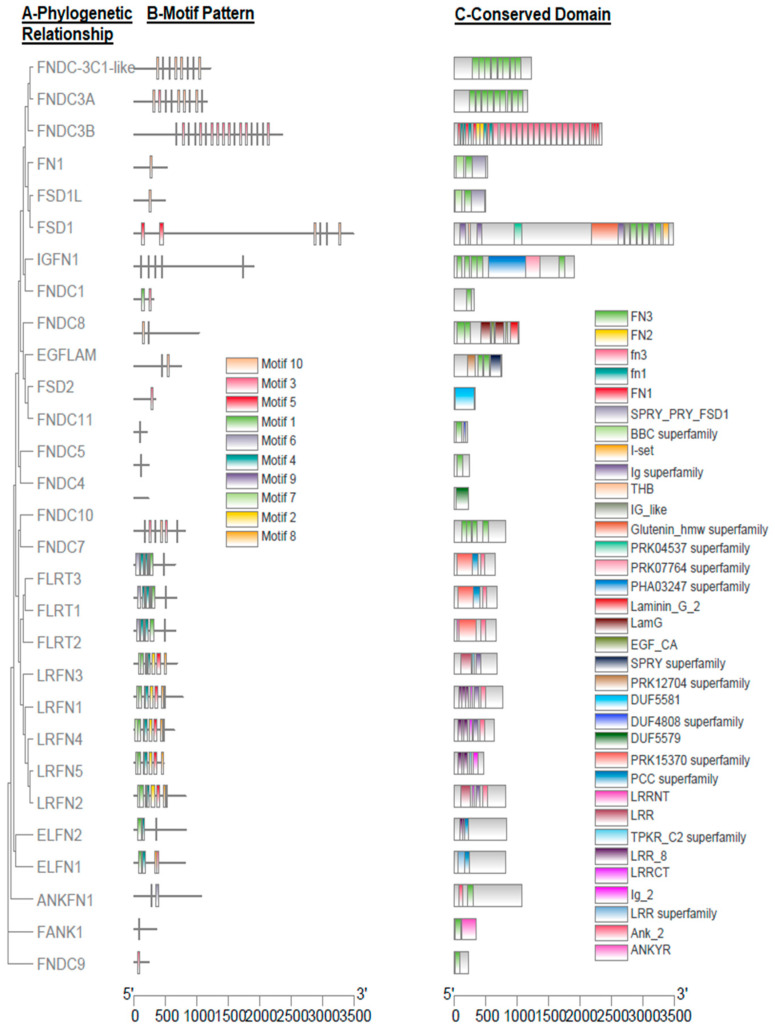
Phylogenetic relationships, motif patterns, and conserved domain regions of buffalo FN-III proteins. (**A**) Phylogenetic relationship of 29 amino acid sequences of FN-III proteins. (**B**) Motif pattern. (**C**) Conserved domain regions. Buffalo ten putative motifs of FN-III proteins are indicated in different colored boxes and details of motifs are enlisted in Table 2.

Furthermore, exon count, phylogenetic relationship, motif pattern, and conserved domain of all the predicted *FN-III* genes in buffalo were explored (Table 1 and Figure 1). The number of exons varied in all genes; as the highest number of exons were prophesied in *FN1*, i.e., 46, while the lowest was in *FNDC9*, i.e., 2 (Table 1). The phylogenetic relationship revealed that all of the buffalo *FN-III* genes were grouped into nine clades, and ANKFN1, FNDC9, and FANK1 were at the base (Figure 1A). In *FN-III* genes, ten putative conserved MEME motifs were observed in buffalo (Figure 1B) and five MEME motifs, including motif 1, 2, 4, 6, and 9 corresponding to 50, 50, 41, 50, and 16 amino acids, respectively, were annotated as Leucine-rich repeat domain, while motif 3 and 10 were annotated as fn3 domain after the Pfams search (Table 2). The CDD BLAST was used to confirm the predicted conserved domains in buffalo *FN-III* genes (Figure 1C). Additionally, the domain of SPRY_PRY_FSD1, BBC, Ig, Glutenin_hmw, PRK04537, PRK07764, PHA03247, Laminin_G_2, LamG, EGF_CA, SPRY, PK12704, DUF5581, DUF4808, DUF5579, PRK15370, PCC, TPKR_C2, Ig_2Ank_2, and ANKYR superfamily has also been dredged up in the buffalo *FN-III* gene family (Figure 1C).

### 3.3. Collinearity Analysis of FN-III Gene Family

**Figure 2:**
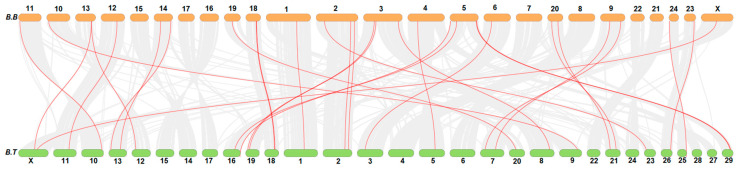
Collinearity analysis of *FN-III* genes family in buffalo (B.B) and *Bos taurus* (B.T).

Collinearity analysis showed that genes of the *FN-III* family in buffalo were distributed over 18 chromosomes, while these genes were present over 21 chromosomes in cattle. Mostly, the buffalo *FN-III* genes were distributed on proximal or terminal ends of the chromosomes as presented in Figure 2.

### 3.4. Structural Configuration of FNDC5 Protein

**Figure 3:**
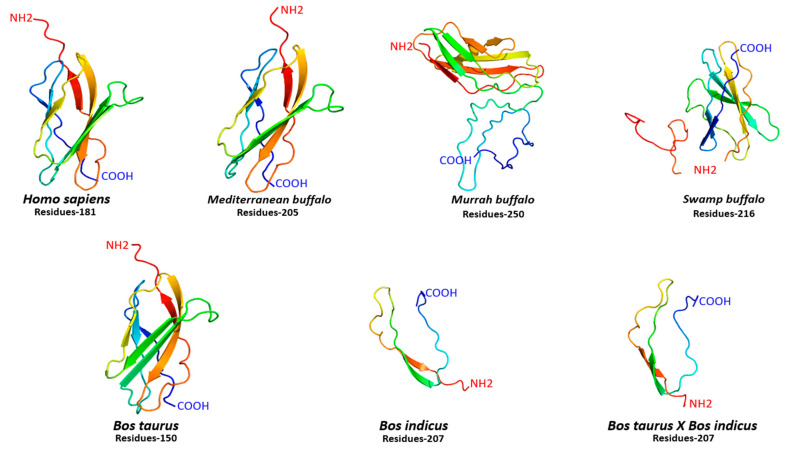
Three-dimensional protein configuration of FNDC5 in humans, cattle, and buffalo.

For comparative structural configuration, three-dimensional protein models for FNDC5 were also predicted in humans, and different buffalo and cattle breeds (Figure 3). It was observed that FNDC5 protein structures in all species varied with a different number of amino acid residues ranging between 181 and 250. Indeed, there was variation in amino acid residues, but the FNDC5 structure in human, *Mediterranean buffalo* and cattle was quite similar to each other (Figure 3). Moreover, secondary structural elements including α-helix, β-sheets, transmembrane helix (TM), and degree of disorder also varied in all species. The α-helix was absent in Murrah buffalo and *Bos taurus*, while human and *Mediterranean buffalo* breeds shared an approximately similar proportion of β-sheets and TM helix (Table S2). Furthermore, protein in cattle was mainly comprised of α-helix and a higher degree of disorder was observed in buffalo breeds (Table S2).

### 3.5. Multiple Sequence Alignment Analysis of Irisin

**Figure 4:**
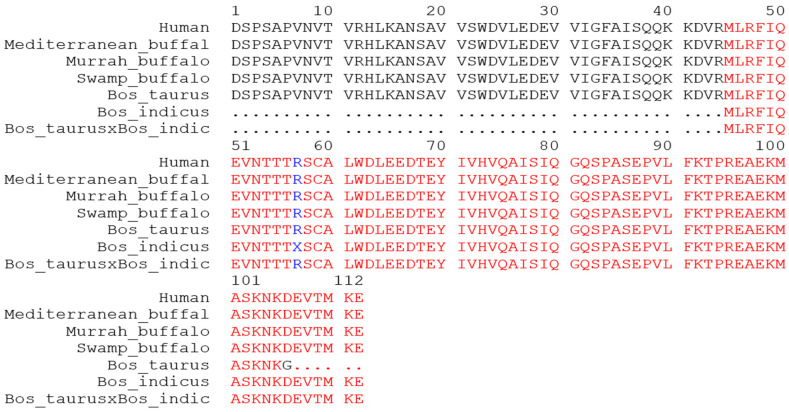
Comparative irisin peptide amino acid analysis of FNDC5 protein in humans, buffalo, and cattle.

The comparative amino acid analysis of irisin peptide revealed conserved nature from human to cattle except for *Bos indicus* and hybrid cattle. *Bos indicus* and hybrid cattle exhibited a long deletion of 44 amino acids toward the NH_2_-terminal end. Only a single amino acid variation D106 > G along with 6 amino acid deletion was also observed in *Bos taurus* at COOH-end. Furthermore, in comparison to humans, all the buffalo breeds had conserved irisin peptide sequences with 100% amino acid sequence homology (Figure 4).

### 3.6. Molecular Docking Analysis of FNDC5/Irisin

**Figure 5:**
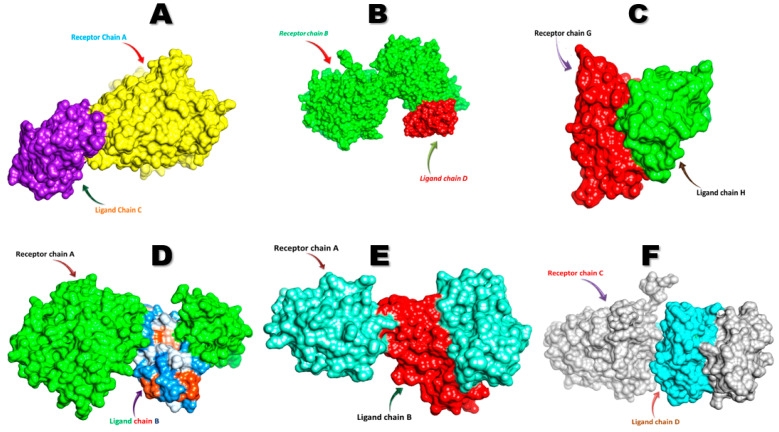
The superimposition of FNDC5 or irisin (ligand) and the receptors (**A**) Androgen (**B**) DDB1 and CUL4 associated factor 6 (**C**) Estrogen-related receptor β (**D**) Estrogen-related receptor γ (**E**) Krüppel-like factor 15 (**F**) Nuclear receptor subfamily 3 group C member 1.

**Figure 6:**
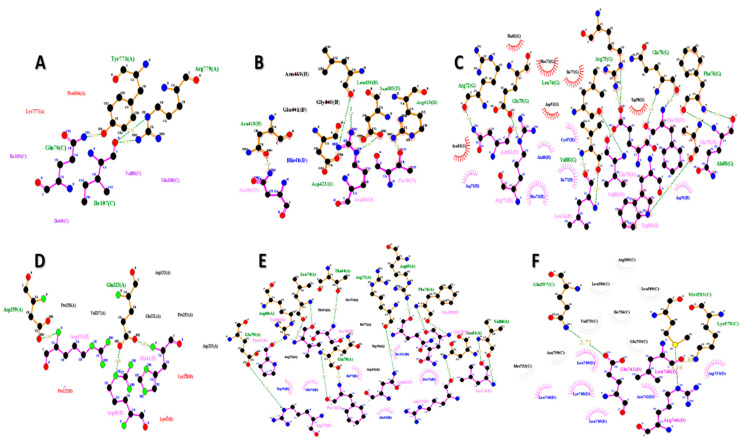
The FNDC5 or irisin (pink; ligand residues) amino acid residues interacting with receptors (**A**) Androgen (**B**) DDB1 and CUL4 associated factor 6 (**C**) Estrogen-related receptor β (**D**) Estrogen-related receptor γ (**E**) Krüppel-like factor 15 (**F**) Nuclear receptor subfamily 3 group C member 1 (all the receptors interacting residues are in green color).

The FNDC5/irisin protein with a molecular weight of 22,869.33 (Dalton) was docked against six receptors to find out the binding affinities. All of the targeted receptors exhibited significant interactions as well as high docking scores ranging from −256.63 to −311.40 (Table 3). A total of 36 hydrogen bonds were detected, which were capable of interacting with the N-terminal portion of all the receptors, except for nuclear receptor subfamily 3 group C member 1 (Table 3, Figure 5 and Figure 6). The FNDC5/irisin also exhibited a strong binding potential with different residues of the selected receptor molecules, where the amino acid residues 36 to 41 were mostly bonded with AR, DCAF6, and ERR-γ (Table 3, Figure 5A,B,D and Figure 6A,B,D). Furthermore, the irisin pocket with amino acid residues ranged between 72 and 91, and showed strong binding potential with ERR-β and KLF15 (Table 3, Figure 5C,E,F and Figure 6C,E,F). The superimposition of FNDC5/irisin (ligand) with all the receptors and their interactions are presented in Figure 5 and Figure 6.

## 4. Discussion

Fibronectin, the glycoprotein, exists in the extracellular matrix (ECM) connecting the collagen fibers with cells. Fibronectins can bind collagen and cell-surface proteins called integrins, which subsequently reorganize the cell’s cytoskeleton and ultimately facilitate the cell movement [30]. These are secreted in inactive and unfolded form by cells, while binding with integrins allows the fibronectin molecules to develop the proteins complex for proper functioning. These protein complexes are composed of two identical monomers bonded by disulfide bond pairs [15]. In vertebrates, the fibronectin is found as soluble plasma fibronectin secreted by the hepatocyte cells of the liver or as insoluble cellular fibronectin, which is the major constituent of ECM, primarily secreted by fibroblasts as soluble dimer, but after the complex cell-mediated process, assembles into an insoluble matrix [15].

### 4.1. The Identification and Characterization of FN-III Gene Family

Fibronectin plays an important role in cell growth, differentiation, migration, and adhesion. Owing to their diversified cellular activities, it also influences vital processes such as embryonic development and wound healing [15]. The fibronectin proteins’ altered expression, organization, and degradation has also been linked with different pathological conditions including fibrosis and cancer [31]. In the present study, a total of 29 buffalo fibronectin genes were identified belonging to the type III domain proteins (Table 1). The conserved motifs analysis elucidated that Leucine-rich repeat and fn3 were the conserved motifs detected in the buffalo *FN-III* gene family (Figure 1 and Table 2).

The members of the FN-III protein are involved in diverse cell functioning through signaling pathways’ as the FNDC5 and FNDC3B promote the cell differentiation of brown fat tissue [32,33]. The FANK1 might regulate apoptosis through JUN and AP-1-mediated transcription activation [34,35]. During cytokinesis, the organization and stability of the microtubule are solely accompanied by the FSD1 protein-associated subset of microtubules [36,37]. Furthermore, the Leucine-rich repeat and fibronectin type III domain such as LRFN1, LRFN2, LRFN3, LRFN4, LRFN5, FLRT1, and FLRT3 proteins are the cell adhesion entities that facilitate homophilic cellular adhesion by a Ca^2+^-independent manner and endorse neurite outgrowth in hippocampal neurons [38,39]. The FLRT3 is essential for fibroblast growth factor-interceded signaling cascades and during embryonic development plays a role in normal morphogenesis [40,41].

The FNDC4 causes the downregulation of pro-inflammatory gene expression by binding to macrophages. It also uses STAT3 activation factor and signaling pathways to inhibit various crucial macrophage activation pathways affecting the macrophage functioning including phagocytosis [42]. The ELFN2 and ELFN1 suppress the phosphatase interactions of the protein phosphatase 1 complex (PP1) [43]. Furthermore, FNDC5A is not only important for spermatid and Sertoli cell development but also has a key role in cell–cell adhesion, which ultimately facilitates the fertilization process in mammals [44].

### 4.2. Structural Configuration of FNDC5 Protein and Its Potential Role in Cellular Activities

Much of the work related to FNDC5 as a messenger initiating adipogenesis in subcutaneous adipose tissue has been documented [45]. Principally, the FNDC5 was detected in peroxisomes and initially defined as an influential factor of skeletal muscle that causes cellular differentiation in mice embryos [46]. Conversely, in another study, weak skeletal muscle signal and strong irisin expression in the embryonic brain of murine were reported [47]. Irisin is secreted from muscle cells with elevated expression of *Ppargc1a* (peroxisome proliferator-activated receptor gamma, coactivator 1 alpha), which encodes the PGC1α (cofactor peroxisome proliferator-activated receptor-γ coactivator 1α) transcript that is involved in various metabolic pathways, specifically the energy metabolism [45,47].

The PGC1α induces the *FNDC5* gene expression and synthesizes an FNDC5 transmembrane protein that comprises 209 amino acids in rats and mice, and 212 amino acids in humans [48]. In the current study, it was observed that FNDC5 protein structures in all representative species (human, buffalo, and cattle) were varied with a variable number of amino acid residues ranging between 181 and 250. Furthermore, irisin, which is a 112-amino-acid-long peptide is conserved in mice and humans, as reported previously [45,49]. Our study agrees with these findings as the buffalo irisin peptide is homologous to humans but variations in the cattle irisin were also detected.

### 4.3. Molecular Docking Analysis of FNDC5/Irisin with Selected Receptors

It is well reported that gonadal function and fertility are strongly associated with metabolic homeostasis, and any disruption could result in crucial aspects to appear, specifically at reproductive age. In fact, irisin can impart a beneficial effect on the body [45] through a complex signaling network acting on several target tissues. The irisin presence in central [50] or peripheral areas such as testes [51] suggests its plausible role in the gonadal-axis through the modulation of some reproductive processes. Thus, in this context, we selected six receptors (including AR, DCAF6, ERR-β, ERR-γ, and KLF15) to unravel their potential bindings with FNDC5/irisin.

#### 4.3.1. FNDC5/Irisin and Estrogen-Related Receptors

Estrogen-related receptors (ERRs) including ER-α and ER-β receptors are the fundamental target sites for the estrogen hormone. These ERRs are mainly distributed over the reproductive organs such as the uterus, ovary, testis, breast, and prostate [52]. In water buffalo, the ERRs are present in the oviduct during both luteal and follicular stages of the estrous cycle and localized in the different parts including ampulla, isthmus, utero-tubal junction, and infundibulum, and are also found in the lamina epithelialis, propria submucosa and tunica of muscularis, and serosa [53]. Similarly, the distribution of ERRs in all layers and regions of the cattle oviduct has been reported in both follicular and luteal phases [54]. In comparison to reproductive organs, the lower expression level of ERR subtypes has also been stated in the livers of females and males [55,56,57]. Additionally, in liver cells, ER-β is less abundant than the ER-α [58,59], while, in metabolic active tissues, the ERRγ is highly expressed during fetal development in the heart, skeletal muscle, and adipose tissues [60].

The ERRγ has shown strong physical interactions with nuclear receptor co-regulators PGC-1 and RIP-140, and the transcription factors of the PGC-1 family strongly stimulate its basal transcriptional activity, but RIP-140 suppresses it [61]. In fact, PGC-1 has a significant role in mitochondrial biogenesis, gluconeogenesis, and thermogenesis, though RIP-140 acts as a repressor of mitochondrial biogenesis and negatively regulates the energy expenditure [62]. Thus, ERRγ and both the co-regulators have an impact on the biological activity as regulators of energy homeostasis [63]. Earlier studies investigated the FNDC5/irisin expression in rat [64] and human [65,66,67] ovaries, in addition to the placenta, testis, pituitary, skeletal and cardiac muscles, brain, and brown adipose tissue [65]. A recent study by Basini et al. has also provided experimental evidence of irisin presence in the pig ovary [68]. The irisin presence or local expression in the follicle of the pig ovary verified its probable regulatory role in the main functional aspect of granulosa cells [68].

Together these observations and the docking scores for ERR-β and ERRγ (−295.57 and −256.63, respectively) in the present study, more specifically, the irisin pocket residues’ binding capabilities, demonstrated that FNDC5/irisin can have a potential physical interaction and, hence, could stimulate the ERRs activity itself or recruit the coactivators, which, ultimately, is helpful in energy homeostasis, cell proliferation activities, and folliculogenesis.

#### 4.3.2. FNDC5/Irisin and Androgen Receptors

Sperm quality and quantity are the attributing factors of male fertility controlled by androgens (5α-dihydrotestosterone and testosterone) facilitated by AR [69]. Androgen receptors belonging to the ligand-dependent nuclear receptors superfamily can initiate spermatogenesis, Sertoli cell proliferation and maturation, development of germ cells, spermatogonia, and spermiogenesis by mediating response through ligand–receptor interaction [69]. Moreover, the DCAF6 is the ligand-dependent nuclear receptor’s coactivator, which could enhance the transcriptional activity of the AR and NR3C1 nuclear receptors. This might act as a substrate receptor for the ubiquitin–protein ligase complex (CUL4-DDB1 E3). It could be involved in protein modifications through the protein ubiquitination pathway, which is subsequently involved in spermatogenesis [70,71]. Our study presented a higher interaction potential of FNDC5/irisin to AR and DCAF6 with docking scores of −311.40 and −256.63, respectively, and the ligand interface residues are Asn36, Thr38, Arg40 and Asn36, Thr38, Arg40, and His41.

#### 4.3.3. FNDC5/Irisin and Glucocorticoid Receptors

The glucocorticoid receptor (GR) is also known as nuclear receptor subfamily 3, group C, member 1 (NR3C1) to which cortisol or other glucocorticoids bind. Almost every cell in the body possesses the GR and controls the gene regulation related to immune response, development, and metabolism. When a hormone or ligand binds to GR, its primary function is to regulate gene transcription [72,73]. The GR in the absence of ligand resides in cytosol and can make an inactive complex with different proteins including heat shock proteins (HSP70 and HSP90) and FK506-binding protein 4 (FKBP4) [74,75,76]. The ligand diffuses into the cytoplasm through the membrane and binds to GR where it dissociates the HSP and develops a ligand–receptor complex. This complex is translocated into the nucleus where it binds to the specific DNA target site and upregulates the gene transcription, while the activated GR complex also prevents the transcriptional binding factors to bind the target DNA site and repress the gene expression [75,76,77].

#### 4.3.4. FNDC5/Irisin and Krüppel-like Factor 15 Receptors

Furthermore, maintenance of muscle mass is mainly dependent on metabolism where the balance exists between catabolism and anabolism [78]. KLF15 is crucial for stimulating gluconeogenesis through the regulation of muscle cell enzymes essential for the catabolism of branched-chain amino acid [79]. Shimizu et al. reported that KLF15 acted downstream of the GR to enhance catabolism and the reduction of skeletal muscle mass [80]. The ligand-mediated GR induction to KLF15 upregulates the ubiquitin–proteasome components (Atrogin-1 and MuRF-1), leading to a boost to the degradation of the cellular protein [80]. Additionally, KLF15 upregulates the proteolytic enzyme gene *BCAT2*, important for the degradation of branched-chain amino acid, which subsequently results in lower intracellular amino acid concentration and inhibits the pro-anabolic activity of the TF mammalian target of rapamycin (mTOR) [80]. The binding affinity of FNDC5/irisin to GR (NR3C1) and KLF15 with a binding score of −308.59 and −260.71, respectively, revealed in the present study, envisaged the potential ability of irisin to regulate the gene expression related to body metabolism and development in buffalo.

In addition to the local expression and presence of irisin in mammals’ ovarian follicles, it has a plausible role in the regulation of the granulosa cells’ functions. Furthermore, the growth of follicular cells should be evaluated through proliferation, nucleic acid synthesis assessment, viability, and metabolic activity interference monitoring through ATP production, which is imperative for follicular development. Taken together, all of the data obtained from the present and previous studies indicates the putative functional and physiological role of FNDC5/irisin peptide in the mammalian reproductive system, particularly in buffaloes. In vivo studies are required to further corroborate these findings and confirm the potential of FNDC5/irisin as a potential candidate for various genomic and nutrigenomic interventions for the improvement of reproductive efficiency in buffalo.

## 5. Conclusions

The present study presented the molecular structure and function of 29 *FN-III* genes, which are widely distributed in the buffalo genome. The phylogenetic relationship, motif, and conserved domain analyses demonstrated that all of the FN-III proteins are evolutionary well conserved with a variety of stable to unstable, hydrophobic to hydrophilic, and thermostable to thermo-unstable proteins naturally existing in buffalo. Comparative structural configuration for FNDC5 predicted variable amino acid residues, but the FNDC5 structure for humans, *Mediterranean buffalo*, and *Bos taurus* are similar to each other. For the first time, we predicted the binding scores and interface residues of FNDC5/irisin as a ligand for six representative receptors, having a functional role in energy homeostasis and significant involvement in folliculogenesis and spermatogenesis in the buffalo.

## Figures and Tables

**Table 1:** Physiochemical properties of the fibronectin gene family in *Bubalus bubalis.*

Gene	Chr.	Exon Count	MW (Da)	A.A	pI	AI	II	GRAVY
Fibronectin 1 (FN1)	2	46	258,641.53	2354	5.28	69.74	40.09	−0.487
Fibronectin type III domain containing 5 (FNDC5)	2	6	22,869.33	205	6.44	92.68	52.30	−0.218
Fibronectin type III domain containing 3B (FNDC3B)	1	31	127,736.34	1160	5.91	69.91	53.98	−0.434
Fibronectin type III and ankyrin repeat domains 1 (FANK1)	23	14	38,413.93	345	8.51	89.51	33.76	−0.334
Fibronectin type III and SPRY domain containing 1 like (FSD1L)	3	16	58,607.09	521	6.32	75.93	46.15	−0.574
Leucine-rich repeat and fibronectin type III domain containing 1 (LRFN1)	18	8	82,023.66	770	7.89	90.16	49.73	−0.066
Leucine-rich repeat and fibronectin type III domain containing 5 (LRFN5)	20	8	52,122.68	466	6.60	95.47	35.44	−0.141
Fibronectin type III and SPRY domain containing 1 (FSD1)	9	13	55,768.58	662	4.96	77.88	48.72	−0.380
Fibronectin type III domain containing 3A (FNDC3A)	13	31	133,632.56	1217	6.44	71.27	46.88	−0.412
Fibronectin type III domain containing 1 (FNDC1)	10	23	205,865.78	1905	9.66	59.01	59.92	−0.799
Leucine-rich repeat and fibronectin type III domain containing 3 (LRFN3)	18	5	72,450.76	679	9.38	87.05	59.78	−0.246
Fibronectin type III and SPRY domain containing 2 (FSD2)	20	15	84,755.73	747	4.81	69.69	47.20	−0.593
Fibronectin type III domain containing 7 (FNDC7)	6	13	85,949.11	811	6.53	77.69	45.18	0.046
Ankyrin repeat and fibronectin type III domain containing 1 (ANKFN1)	3	20	120,567.79	1068	6.52	80.73	58.15	−0.467
Immunoglobulin like and fibronectin type III domain containing 1 (IGFN1)	5	26	347,525.99	3490	6.49	55.35	34.98	−0.590
Fibronectin type III domain containing 4 (FNDC4)	12	7	24,753.16	230	7.66	88.87	55.08	−0.252
Fibronectin type III domain containing 8 (FNDC8)	3	4	34,298.93	312	5.29	80.93	46.44	−0.370
Leucine-rich repeat and fibronectin type III domain containing 4 (LRFN4)	5	3	66,839.10	636	6.70	94.14	42.55	−0.028
Fibronectin type III domain containing protein 3C1-like (LOC102393884)	X	27	157,320.54	1433	6.79	71.84	45.92	−0.439
Fibronectin leucine-rich transmembrane protein 2 (FLRT2)	11	4	73,773.40	660	7.89	94.18	36.58	−0.185
EGF like, fibronectin type III and laminin G domains (EGFLAM)	19	23	112,751.54	1032	6.53	74.46	41.70	−0.325
Fibronectin type III domain containing 9 (FNDC9)	9	2	25,342.98	227	5.65	85.99	54.56	−0.055
Leucine-rich repeat and fibronectin type III domain containing 2 (LRFN2)	2	2	87,694.08	820	6.59	90.88	44.73	−0.097
Fibronectin leucine-rich transmembrane protein 3 (FLRT3)	14	3	73,171.75	649	7.56	94.18	44.53	−0.296
Fibronectin leucine-rich transmembrane protein 1 (FLRT1)	5	2	74,144.68	677	6.15	96.88	32.12	−0.122
Fibronectin type III domain containing 11 (FNDC11)	14	4	38,198.37	333	6.81	96.34	53.23	−0.280
Fibronectin type III domain containing 10 (FNDC10)	5	3	24,097.32	225	9.11	87.33	66.15	0.124
Extracellular leucine-rich repeat and fibronectin type III domain containing 2 (ELFN2)	4	4	90,363.67	824	7.78	81.78	48.76	−0.295
Extracellular leucine-rich repeat and fibronectin type III domain containing 1 (ELFN1)	24	3	87,687.60	808	8.82	79.43	61.89	−0.351

[Chr (Chromosome), MW (Molecular Weight in Daltons), A.A (number of amino acids), pI (Isoelectric point), AI (Aliphatic Index), II (Instability Index), and GRAVY (Grand Average of hydropathicity Index)].

**Table 2:** Ten differentially conserved motifs detected in *FN-III* genes family in Buffalo.

Motif	Protein Sequence	Length	Pfam Domain
MEME-1	DNFIAAIPRRDFANMTGLVDLTLSRNTISHIEAGAFDDLENLRALHLDNN	50	LRR_8
MEME-2	NPLHCNCELLWLRRLAREDDLETCASPPGLTGRYFWSVPEEEFLCEPPLI	50	LRRCT
MEME-3	LTNLEPDTTYRLCVQALNSAG	21	fn3
MEME-4	MVNLETLRLDHNLIDTIPPGAFSELHKLARLDLTSNRLQKL	41	LRR_8
MEME-5	HWVAPDGRLVGNSSRTRVYPNGTLDILITTSGDSGAFTCIASNAAGEATA	50	I-set
MEME-6	CPSVCRCDRGFIYCNDRGLTSIPAGIPEDATTLYLQNNQINNAGIPADLK	50	LRRNT
MEME-7	CPKRCICQNLSPSLSTLCAKKGLLFVPPNIDRRTVELRL	39	Toxin_11
MEME-8	WPVQRPAPGIRMYQIQYNSSADDTLVYRM	29	-
MEME-9	LEDLDLSYNNLESIPW	16	LRR_4
MEME-10	GTEYRFRVRACNEAGEGPLSEPYTVTTPP	29	fn3

[LRR_8, Leucine-rich repeat; LRRCT, Leucine-rich repeat C-terminal domain; fn3, Fibronectin type III domain; I-set, Immunoglobulin I-set domain; LRRNT, Leucine-rich repeat N-terminal domain; Toxin_11, Spasmodic peptide gm9a conotoxin from Conus species; LRR_4, Leucine-rich repeats (2 copies)].

**Table 3:** Molecular docking results of ligand (FNDC5 or Irisin) binding affinity with different receptors.

Sr. No.	Receptor	Docking Score	Ligand RMSD (A0)	Ligand Interacting Residues
1	Androgen	−311.40	86.42	Asn36, Thr38, Arg40
2	DDB1 and CUL4 associated factor 6	−256.63	79.76	Asn36, Thr38, Arg40, His41
3	Estrogen-related receptor β	−295.57	108.96	Arg72, Mse73, Leu74, Arg75, Phe76, Ile77, Gln78, Glu79, Val80, Asn81, Cys87, Ala88, Trp90, Asp91
4	Estrogen-related receptor γ	−256.63	79.76	Arg40, His41, Lys43, Lys120, Pro122, Arg123
5	Krüppel-like factor 15	−260.71	81.85	Ser30, Pro31, Arg72, Mse73, Leu74, Arg75, Phe76, Ile77, Gln78, Glu79, Asn81, Ala88, Trp90, Gln108, Pro112, Val180
6	Nuclear receptor subfamily 3 group C member 1	−308.59	108.34	Lys740, Glu741, Asn742, Leu744, Leu745, Arg746, Leu748, Leu749, Asp753

## Data Availability

Not applicable.

## Supplementary Materials

The following are available online at https://www.mdpi.com/article/10.3390/biology10111207/s1, Table S1: The accession number of FN-III protein sequences used in this study, Table S2: Secondary structure and disorder prediction of FNDC5 protein.
