# Supplementary material for: Comparative Genomic Characterization of Buffalo Fibronectin Type III Domain Proteins: Exploring the Novel Role of FNDC5/Irisin as a Ligand of Gonadal Receptors
Source: Biology (Basel). 2021 Nov 19;10(11):1207. doi: 10.3390/biology10111207 (PMC8615036; doi:10.3390/biology10111207)
Supplement: Supplementary file 1 [file biology-10-01207-s001.zip › biology-1394392-supplementary.pdf]

biology

MDPI

Article

Comparative Genomic Characterization of Buffalo Fibronectin Type III Domain Proteins: Exploring the Novel Role of FNDC5/Irisin as a Ligand of Gonadal Receptors

Siwen Wu [1,†], Faiz-ul Hassan [2,†], Yuhong Luo [1], Israr Fatima [3], Ishtiaq Ahmed [4], Awais Ihsan [5], Warda Safdar [6], Qingyou Liu [1,*] and Saif ur Rehman [1,*]

1 State Key Laboratory for Conservation and Utilization of Subtropical Agro-Bioresources, Guangxi University, Nanning 530005, China; siwenwu123@163.com (S.W.); luoyuhong0720@163.com (Y.L.)
2 Institute of Animal and Dairy Sciences, University of Agriculture, Faisalabad 38040, Pakistan; f.hassan@uaf.edu.pk
3 Department of Bioinformatics and Biotechnology, Govt. College University, Faisalabad 38000, Pakistan; fatimaisrar926@gmail.com
4 School of Medical Science, Gold Coast Campus, Griffith University, Southport, QLD 4222, Australia; ishtiaq.ahmed@griffithuni.edu.au
5 Department of Biosciences, COMSATS University Islamabad, Sahiwal Campus, Sahiwal 57000, Pakistan; awais@cuisahiwal.edu.pk
6 Department of Biochemistry, Bahauddin Zakariya University, Multan 60000, Pakistan; wardakhanw9@gmail.com
* Correspondence: qyliu-gene@gxu.edu.cn (Q.L.); Saif_ali28@yahoo.com (S.u.R.)
† These authors contributed equally.

Citation: Wu, S.; Hassan, Fu.; Luo, Y.; Fatima, I.; Ahmed, I.; Ihsan, A.; Safdar, W.; Liu, Q.; Rehman, S.u. Comparative Genomic Characterization of Buffalo Fibronectin Type III Domain Proteins: Exploring the Novel Role of FNDC5/Irisin as a Ligand of Gonadal Receptors. *Biology* **2021**, *10*, 1207. https://doi.org/10.3390/biology10111207

Academic Editor: Hiroetsu Suzuki

Received: 9 September 2021
Accepted: 16 November 2021
Published: 19 November 2021

Publisher's Note: MDPI stays neutral with regard to jurisdictional claims in published maps and institutional affiliations.

Copyright: © 2021 by the authors. Licensee MDPI, Basel, Switzerland. This article is an open access article distributed under the terms and conditions of the Creative Commons Attribution (CC BY) license (http://creativecommons.org/licenses/by/4.0/).

Table S1. The accession number of FN-III protein sequences used in this study.

Gene	Human	Cattle	Buffalo
Fibronectin 1 (FN1)	XP_005246465.1	XP_005202842.1	XP_025134000.1
Fibronectin type III domain containing 5 (FNDC5)	NP_001165411.2	NP_001098891.1	XP_025134275.1
Fibronectin type III domain containing 3B (FNDC3B)	NP_001128567.1	XP_005201753.1	XP_025140051.1
Fibronectin type III and ankyrin repeat domains 1 (FANK1)	NP_001337868.1	XP_024841256.1	XP_025129687.1
Fibronectin type III and SPRY domain containing 1 like (FSD1L)	NP_001138785.1	XP_024851352.1	XP_006061602.1
Leucine-rich repeat and fibronectin type III domain containing 1 (LRFN1)	NP_065913.1	XP_024834841.1	XP_006068730.1
Leucine rich repeat and fibronectin type III domain containing 5 (LRFN5)	NP_001317035.1	XP_005222160.1	XP_025127666.1
Fibronectin type III and SPRY domain containing 1 (FSD1)	NP_001317358.1	NP_001074987.1	XP_006042571.1
Fibronectin type III domain containing 3A (FNDC3A)	NP_001073141.1	XP_010808981.1	XP_025119024.1
Fibronectin type III domain containing 1 (FNDC1)	NP_115921.2	XP_024852897.1	XP_006051319.2
Leucine rich repeat and fibronectin type III domain containing 3 (LRFN3)	NP_078785.1	NP_001070427.1	XP_025125629.1
Fibronectin type III and SPRY domain containing 2 (FSD2)	NP_001007123.1	NP_001192937.1	XP_025127513.1
Fibronectin type III domain containing 7 (FNDC7)	NP_001138409.1	XP_024845811.1	XP_006052208.2
Ankyrin repeat and fibronectin type III domain containing 1 (ANKFN1)	NP_001352687.1	XP_027373748.1	XP_006076307.1
Immunoglobulin like and fibronectin type III domain containing 1 (IGFN1)	NP_001158058.1	XP_024832341.1	XP_025141009.1
Fibronectin type III domain containing 4 (FNDC4)	XP_038969018.1	NP_001095794.1	XP_006046222.1
Fibronectin type III domain containing 8 (FNDC8)	NP_060029.1	NP_001069912.1	XP_025136871.1
Leucine-rich repeat and fibronectin type III domain containing 4 (LRFN4)	NP_001350453.1	NP_001193183.1	XP_025142533.1
Fibronectin type III domain containing protein 3C1-like (LOC102393884)	XP_038956079.1	XP_019811022.1	XP_025131684.1
Fibronectin leucine rich transmembrane protein 2 (FLRT2)	NP_001333072.1	XP_003586651.1	XP_006069515.1

EGF like, fibronectin type III and laminin G domains (EGFLAM)	NP_001192230.1	NP_001076947.1	XP_025126375.1
Fibronectin type III domain containing 9 (FNDC9)	NP_001001343.2	XP_005209734.1	XP_006075933.1
Leucine-rich repeat and fibronectin type III domain containing 2 (LRFN2)	NP_065788.1	NP_001179524.1	XP_025126259.1
Fibronectin leucine rich transmembrane protein 3 (FLRT3)	NP_037413.1	NP_001179603.1	XP_006051375.1
Fibronectin leucine rich transmembrane protein 1 (FLRT1)	NP_001371395.1	XP_005227270.1	XP_006050918.2
Fibronectin type III domain containing 11 (FNDC11)	NP_001306081.1	XP_024856577.1	XP_025119638.1
Fibronectin type III domain containing 10 (FNDC10)	NP_001229588.1	XP_024832575.1	XP_025141526.1
Extracellular leucine-rich repeat and fibronectin type III domain containing 2 (ELFN2)	NP_443138.2	XP_005206935.1	XP_025139285.1
Extracellular leucine-rich repeat and fibronectin type III domain containing 1 (ELFN1)	NP_001122108.1	XP_005225286.2	XP_025130646.1

Table S2. Secondary structure and disorder prediction of FNDC5 protein.

	**Disordered**	**Alpha helix**	**Beta strand**	**TM helix**
Homo sapiens	35%	08%	42%	10%
Mediterranean buffalo	46%	11%	39%	10%
Murrah buffalo	44%	0%	48	08%
Swamp buffalo	48%	07%	40%	09%
Bos taurus	39%	0%	50%	-
Bos indicus	40%	26%	21%	18%
Bos taurus X Bos indicus	40%	26%	21%	18%

[TM: transmembrane helix].
